# Randomized Prospective Study Comparing Wide-Awake Local Anesthesia No Tourniquet (WALANT) and Locoregional Anesthesia in Minimally Invasive Hallux Valgus Surgery: Preliminary Results

**DOI:** 10.1177/24730114261455303

**Published:** 2026-07-16

**Authors:** Daniele Marcolli, Alice Montagna, Ivan Pichierri, Tommaso Forin Valvecchi, Mirko Colombo, Riccardo Chierichini, Federico Alberto Grassi, Alessio Bernasconi, Pietro S. Randelli

**Affiliations:** 1 Università Degli Studi di Milano, Milan, Italy; 2 U.O.S.D. Centro Patologia del Piede, ASST Gaetano Pini-CTO, Milan, Italy; 3 Clinica Ortopedica e Traumatologica, Fondazione IRCCS Policlinico San Matteo, Pavia, Italy; 4 Department of Anaesthesia and Intensive Care, Gaetano Pini Hospital, Milan, Italy; 5 Department of Clinical, Surgical, Diagnostic, and Pediatric Sciences, University of Pavia, Pavia, Italy; 6 Department of Public Health, Orthopaedic, and Traumatology Unit, University Federico II of Naples, Naples, Italy; 7 ASST Centro Specialistico Ortopedico Traumatologico Gaetano Pini-CTO, 1st Orthopaedic Clinic, Milan, Italy; 8 Laboratory of Applied Biomechanics, Department of Biomedical Sciences for Health, Università Degli Studi di Milano, Milan, Italy; 9 Department of Biomedical, Surgical, and Dental Sciences, Università Degli Studi di Milano, Milan, Italy; 10 Research Center for Adult and Pediatric Rheumatic Diseases (RECAP-RD), Università Degli Studi di Milano, Milan, Italy

**Keywords:** hallux valgus, minimally invasive surgery, MICA technique, WALANT, wide-awake anesthesia, locoregional anesthesia, foot surgery, pain assessment

## Abstract

**Background:**

Minimally invasive techniques for hallux valgus correction, such as minimally invasive chevron Akin (MICA), are increasingly adopted and traditionally performed under locoregional anesthesia (LRA). The choice of anesthetic technique may influence perioperative outcomes, patient experience, and resource utilization. The Wide-Awake Local Anesthesia No Tourniquet (WALANT) technique has recently been introduced in foot surgery as a potential alternative.

**Methods:**

A prospective, randomized, single-center study was conducted on 40 patients with mild to moderate hallux valgus undergoing correction with the MICA technique. Patients were randomized into 2 groups: LRA (n = 20) and WALANT (n = 20). The primary endpoint of this study was the assessment and comparison of intraoperative and postoperative pain at 24 hours between the WALANT and LRA groups. Secondary endpoint was the comparison of anesthetic time and recovery stay between the 2 techniques.

**Results:**

Mean intraoperative and postoperative NRS pain scores showed no statistically significant differences between the 2 groups. The anesthesia time and the recovery stay were significantly shorter in the WALANT group compared with the LRA group. All WALANT procedures were successfully completed without anesthetic conversion.

**Discussion:**

The application of WALANT in hallux valgus surgery meets the growing demand for efficient perioperative pathways, potentially improving operating room efficiency and reducing hospitalization time. In this preliminary study, no statistically significant difference in intraoperative or postoperative pain at 24 hours was detected between WALANT and LRA with the numbers available. These findings suggest that WALANT may represent a feasible anesthetic approach for MICA correction of hallux valgus.

**Conclusion:**

In this prospective randomized study, no statistically significant difference in intraoperative or postoperative pain was detected between WALANT and locoregional anesthesia with the numbers available. WALANT was associated with shorter anesthesia time and recovery stay, suggesting that it may represent a feasible anesthetic option for MICA hallux valgus surgery. Further studies with larger cohorts are required to confirm these findings.

**Level of Evidence:**

Level II, prospective randomized trial.

## Introduction

Hallux valgus is one of the most common forefoot deformities, affecting a significant proportion of the adult population and often impairing quality of life.^1^ The condition is characterized by lateral deviation of the hallux and medial deviation of the first metatarsal, leading to pain, difficulty with footwear, and functional limitations. When conservative measures fail, surgical correction is frequently indicated.^2^ Minimally invasive techniques for hallux valgus correction, including minimally invasive chevron Akin (MICA), have gained increasing popularity in recent years.^3,4^ Clinical and radiographic studies have reported satisfactory angular correction and high patient satisfaction with this approach.^5,6^ Traditionally, this procedure is performed under locoregional anesthesia (LRA), which provides adequate pain control but still requires anesthesia personnel, monitoring, and postoperative observation.^7^

Given the high volume of hallux valgus corrections performed worldwide, techniques that allow for efficient, safe, and well-tolerated anesthesia are particularly valuable. The wide-awake local anesthesia no tourniquet (WALANT) technique, although defined many years ago, has become widely adopted in hand surgery and is now a standard practice in many centers.^8-11^

WALANT technique implies injection of a mixture of local anesthetic (like lidocaine) and a vasoconstrictor (like epinephrine) directly into the surgical site. The procedure is performed without the use of tourniquet in the awake patient allowing the surgeon to communicate with the patient himself.^9,12,13^

Its major advantages include the reduction of anesthetic risks, operative costs, and hospital stay compared with general or spinal anesthesia.^14-16^ These benefits gained particular relevance during the COVID-19 pandemic, when elective surgeries performed under traditional anesthesia were often postponed, highlighting the need for safe and efficient alternatives that minimize hospitalization time.^17,18^

Although WALANT has been extensively validated in hand and upper limb surgery, its application in lower extremity procedures remains limited. Few reports have demonstrated its feasibility for traumatic conditions such as ankle fractures and Achilles tendon repair, but evidence regarding its use in elective foot surgery is still scarce.^19,20^

Given the growing interest in outpatient, resource-efficient surgical pathways, WALANT may represent a safe, cost-effective, and patient-centered alternative for hallux valgus correction.^16,21^

The aim of this study was to compare intraoperative and postoperative pain (at 24 hours) between patients who underwent to MICA osteotomy with the WALANT technique with those with LRA technique. Secondary endpoint was to compare WALANT and LRA in terms of surgical times and hospital stay.

## Materials and Methods

A prospective, randomized, single-center study was conducted on 40 consecutive patients affected by mild to moderate hallux valgus who underwent surgical correction using the MICA technique between May 2025 and October 2025. Patient enrollment was performed according to predefined inclusion and exclusion criteria. Inclusion criteria comprised an age between 18 and 75 years, the presence of clinically symptomatic hallux valgus confirmed by radiographic evaluation, and failure of conservative treatment, including orthotic management and footwear modification, for a minimum duration of 6 months. Exclusion criteria included previous forefoot surgery on the affected side, inflammatory arthropathies, diabetes mellitus associated with peripheral neuropathy, known coagulation disorders, or inability to provide informed consent. Patients with diabetes, known coagulation disorders, vascular insufficiency, or active infections were excluded to minimize confounding factors and reduce the risk of complications.

Before surgery, patients were randomized using a block randomization method with a 1:1 allocation ratio and assigned to one of 2 groups based on the anesthetic technique employed. Group 1 received LRA and included 20 patients, whereas Group 2 received WALANT, also including 20 patients.

Baseline demographic and clinical data were collected for all participants, including age, sex, body mass index (BMI), and operated side, in order to ensure comparability between groups and to assess the effectiveness of the randomization process.

The primary endpoint of the study was the assessment and comparison of intraoperative pain and postoperative pain at 24 hours between the WALANT and LRA groups. Twenty-four-hour postoperative pain scores were collected after discharge via standardized telephone follow-up performed by the study team. Postoperative analgesic therapy was standardized for all patients, and no differences in type or dosage of rescue analgesic medication were observed between the 2 groups at 24 hours. No supplemental local anesthetic injections were required or administered intraoperatively in the WALANT group.

Pain intensity was measured using the Numeric Rating Scale (NRS), a validated 11-point scale ranging from 0, indicating no pain, to 10, indicating the worst imaginable pain. Intraoperative pain, defined as the average overall pain reported by the patient during the surgical procedure, was recorded immediately after completion of the surgical procedure, whereas postoperative pain was assessed at 24 hours following surgery.

Secondary endpoints included a comparison between the WALANT and LRA techniques in terms of anesthesia-related operative times and postoperative recovery stay, with the aim of evaluating efficiency and perioperative workflow optimization associated with each anesthetic approach.

### Anesthetic Techniques

For patients allocated to the WALANT group, a total volume of 30 mL of anesthetic solution was prepared and administered.

The solution consisted of 1% lidocaine buffered with 8.4% sodium bicarbonate in a 10:1 ratio, without the addition of a vasoconstrictor such as epinephrine, in order to optimize anesthetic efficacy and reduce injection-related discomfort.

The anesthetic was carefully infiltrated along the planned skin incision and circumferentially around the first metatarsal, ensuring adequate diffusion to both superficial and deep tissues involved in the surgical procedure. Local anesthesia in the WALANT group targeted the superficial and deep branches of the medial and lateral plantar nerves as well as the dorsal digital nerves, ensuring complete coverage of the operative field for minimally invasive hallux valgus correction. Infiltration was performed using established anatomical landmarks to accurately target both superficial and deep layers of the plantar nerves, ensuring complete coverage of the operative field.

No pneumatic tourniquet was applied at any stage of the operation. All WALANT procedures in the present study were performed by surgeons with experience in the technique and following a standardized protocol, thereby minimizing operator-dependent variability. The procedures were performed in a operating room by the surgeon and a scrub technician, with the presence of an anesthesiologist.

Before incision, patients in the WALANT group were tested to confirm complete anesthesia; no additional local anesthetic was required during the procedure.

In the LRA group, anesthesia was achieved through an ultrasound-guided popliteal sciatic nerve block performed under sterile conditions by an experienced anesthesiologist. Using a high-frequency linear probe, the sciatic nerve was identified in the popliteal fossa just above the bifurcation into tibial and common peroneal nerves. A lateral-to-medial in-plane needle approach was used, advancing under direct visualization toward the nerve. A 1:1 mixture of 2% mepivacaine and 1% ropivacaine was administered, with a total volume of 15 to 20 mL depending on patient anatomy and real-time spread of the anesthetic around the nerve. After injection, the block latency was monitored for 20 to 30 minutes, confirming sensory loss to pinprick and cold test, as well as motor block assessed by plantarflexion and dorsiflexion of the ankle. Adequate blockade was confirmed before skin incision to ensure sufficient anesthetic effect for the procedure.

All patients in the LRA group received the same anesthetic protocol, consisting of mild intravenous sedation with 2 mg midazolam, while maintaining spontaneous breathing and the ability to cooperate during surgery. No tourniquet was used in the LRA group.

Patients in the LRA group underwent surgery in a standard operating room with an anesthesiologist present throughout the procedure.

### Surgical Procedure

All surgical procedures were performed by 2 experienced foot and ankle surgeons who were not involved in the preoperative or postoperative clinical evaluation of the patients, in order to minimize assessment bias. Hallux valgus correction was carried out using the MICA technique, following the standardized operative steps previously described in the literature.^4,3^

Patients were positioned in the supine position with both feet overhanging the distal end of the operating table. This positioning allowed unobstructed access to the operative foot, facilitated optimal placement of the limb under the C-arm fluoroscopy unit, and enabled free intraoperative manipulation of the foot in multiple planes. All surgical steps were performed under continuous image-intensifier guidance to ensure accurate osteotomy execution, controlled fragment displacement, and appropriate implant positioning.

A 3-mm skin incision was made on the dorsomedial aspect of the first metatarsal head at the base of the medial eminence using a beaver blade. Through this minimal incision, dedicated periosteal elevators were introduced to carefully release the medial capsule and expose the bony surface while preserving surrounding soft tissues. The distal chevron osteotomy of the first metatarsal was then performed using a Shannon burr with a diameter of 2 mm and a length of 20 mm, which was introduced through the previously created incision.

Under fluoroscopic control, the burr was oriented perpendicular to the longitudinal axis of the first metatarsal in the transverse plane, with a slight distal inclination when required, and directed plantarly at approximately 30° relative to the coronal plane of the bone. The dorsal cut of the chevron osteotomy was performed first, followed by completion of the plantar cut. The plantar limb of the osteotomy was created by delicately advancing the burr from its initial position while guiding the surgeon’s hand toward the tip of the hallux, ensuring a controlled and reproducible chevron configuration.

Once the osteotomy was completed, controlled lateral displacement of the metatarsal head was achieved using a periosteal elevator, allowing correction of the deformity. Stable internal fixation was obtained using 2 specifically designed cannulated compression screws (Arthrex), which were inserted through proximal medial stab incisions. Screw placement was performed under fluoroscopic guidance with the goal of achieving a stable 3-point fixation engaging the cortical bone of the first metatarsal.

Following fixation, the medial bony spicula generated by the lateral translation of the metatarsal head were removed using a Shannon burr measuring 2 × 12 mm. Additional refinement of the medial eminence was performed through the initial incision using a 3.1-mm wedge burr, allowing removal of residual medial bone and completion of the metatarsal head contouring.

Finally, a percutaneous Akin osteotomy of the proximal phalanx was performed when indicated. This was achieved using a Shannon burr measuring 2 × 12 mm, introduced through a 2-mm medial skin incision at the level of the proximal phalanx. The osteotomy was completed under fluoroscopic control, and stable fixation was obtained using a single cannulated compression screw (MicroAcutrak, Acumed). Final fluoroscopic images were obtained to confirm adequate correction, implant positioning, and overall alignment.

### Perioperative Management

All patients received identical preoperative antibiotic prophylaxis (single-dose intravenous cefazolin 2 g), as none had a known allergy to first-generation cephalosporins, and standardized postoperative care, including dressing, weight-bearing protocol, and pain management.

Postoperatively, patients were transferred to their hospital room and monitored by ward nursing staff until clinical evaluation and discharge by the surgeon.

Patients were considered suitable for discharge following evaluation by the operating surgeon or a member of the surgical team, once full recovery from anesthesia was achieved, and when stable vital signs, adequate pain control (NRS ≤ 3), ability to ambulate safely, and absence of immediate postoperative complications were confirmed, in accordance with institutional postoperative discharge criteria.

All patients were discharged with a standardized analgesic regimen consisting of paracetamol 1000 mg 3 times daily, ketoprofen 80 mg twice daily, and tramadol hydrochloride (15 drops as rescue medication in case of uncontrolled pain), with identical postoperative prescriptions in both groups.

### Statistical Analysis

Categorical variables were described in the respective tables with absolute values and percentages, whereas continuous variables were described differently according to the type of distribution. Variables with a normal distribution were described with mean and SD; variables that did not have a normal distribution were described with median and IQR (Q1-Q3).

The Shapiro-Wilk normality test was used to evaluate the normal distribution of the sample. Variables with a Gaussian distribution were analyzed with the Student *t* test, whereas variables without a Gaussian distribution were analyzed with the Mann-Whitney *U* test for independent samples and with the Wilcoxon test for dependent samples. Dichotomous variables were analyzed with the χ^2^ test. Level l of significance was set at <.05. CIs (95%) for NRS pain scores were calculated using a parametric method based on SEs estimated from the linear mixed effects model.

All statistical analyses were performed using IBM SPSS Statistics for Windows, version 29.0 (IBM Corp).

No formal sample size or statistical power calculation was performed, as this is a preliminary study conducted on a small patient cohort.

### Ethical Considerations

All procedures performed in this study involving human participants were in accordance with the ethical standards of the Declaration of Helsinki and with the principles of good clinical practice. Informed consent was obtained from all individual participants included in the study. The study was approved by the Institutional Scientific Board of the institution and also received approval from the Ethics Committee (Approval No. 237/2023, AOU FED II).

## Results

The baseline characteristics of the study population are summarized in Table 1. The 2 groups were well matched at baseline, with no statistically significant differences observed with respect to age, BMI, sex distribution, or operated side, confirming the effectiveness of the randomization process. The mean age was 50.4 ± 12.9 years, ranging from 30 to 81 years, in the WALANT group and 52.7 ± 13.0 years, ranging from 22 to 68 years, in the LRA group (*P* = 0.58). Mean BMI values were also comparable between groups, measuring 25.3 ± 3.2 (range 20.3-32.4) in the WALANT group and 25.0 ± 3.4 (range 19.3-32.0) in the LRA group (*P* = 0.78).

**Table 1.** Demographic Data of WALANT and LRA Groups.

Characteristic	Group 1 (WALANT)	Group 2 (LRA)	*P* Value
No. of patients	20	20	
Age, y, mean ± SD (range)	50.4 ± 12.9 (30-81)	52.7 ± 13.0 (22-68)	.58
BMI, mean ± SD (range)	25.3 ± 3.2 (20.3-32.4)	25.0 ± 3.4 (19.3-32.0)	.78
Gender, n (%)			.45
Male	6 (30)	3 (15)	
Female	14 (70)	17 (85)	
Operated side, n (%)			>.99
Right	11 (55)	12 (60)	
Left	9 (45)	8 (40)	

Abbreviations: BMI, body mass index; LRA, locoregional anesthesia; WALANT, wide-awake local anesthesia no tourniquet.

Sex distribution did not differ significantly between groups, with males accounting for 30% and females for 70% of patients in the WALANT group, compared with 15% males and 85% females in the LRA group (*P* = .45). Similarly, the laterality of the procedure was evenly distributed, with right-sided surgery performed in 55% of cases in the WALANT group and 60% in the LRA group, and left-sided surgery in 45% and 40% of cases, respectively (*P* > .99). Overall, no statistically significant differences were identified in baseline demographic variables between the 2 groups.

Intraoperative pain levels were low in both groups. The mean intraoperative NRS score was 0.84 ± 1.46, with a range from 0 to 6, in the WALANT group (95% CI 0.36-1.32) and 0.64 ± 1.26, with a range from 0 to 5, in the LRA group (95% CI 0.14-1.14). No statistically significant difference in intraoperative pain perception was observed between the 2 anesthetic techniques (*P* = .65).

At 24 hours postoperatively, pain levels increased as expected but remained comparable between groups. The mean postoperative NRS score at 24 hours was 3.0 ± 1.45, ranging from 1 to 6, in the WALANT group (95% CI 2.52-3.48) and 2.79 ± 1.71, also ranging from 1 to 6, in the LRA group (95% CI 2.11-3.47), with no statistically significant difference between groups (*P* = 0.68; Table 2).

**Table 2.** Distribution of Intraoperative and Postoperative NRS Pain Scores.

	Intraoperative		Postoperative	
Pain Score	LRA, n (%) (n = 20)	WALANT, n (%) (n = 20)	LRA, n (%) (n = 20)	WALANT, n (%) (n = 20)
0	14 (70.0)	12 (60.0)	2 (10.0)	1 (5.0)
1	3 (15.0)	4 (20.0)	3 (15.0)	2 (10.0)
2	2 (10.0)	3 (15.0)	4 (20.0)	5 (25.0)
3	0 (0)	0 (0)	4 (20.0)	5 (25.0)
4	0 (0)	0 (0)	2 (10.0)	3 (15.0)
5	1 (5.0)	0 (0)	2 (10.0)	3 (15.0)
6	0 (0)	1 (5.0)	3 (15.0)	1 (5.0)

Abbreviations: LRA, locoregional anesthesia; NRS, Numeric Rating Scale; WALANT, wide-awake local anesthesia no tourniquet.

In contrast, significant differences were observed with regard to anesthesia-related efficiency outcomes. The mean anesthesia time was significantly shorter in the WALANT group, averaging 3.6 ± 0.5 minutes (95% CI 3.44-3.76), compared with 8.4 ± 2.0 minutes in the LRA group (95% CI 7.61-9.19), demonstrating a highly significant difference (*P* < .0001). Furthermore, patients treated under WALANT anesthesia experienced a significantly shorter postoperative recovery stay, with a mean duration of 9.45 ± 0.80 hours (95% CI 9.19-9.71), compared with 15.15 ± 8.41 hours in the LRA group (95% CI 11.82-18.48) (*P* = 0.004), as reported in Table 3.

**Table 3.** Comparison Between the Mean Anesthesia Time, Intraoperative Pain, Postoperative Pain and Recovery Stay Between the WALANT and LRA Groups.

Outcome	Group 1 (WALANT), Mean ± SD (Range)	Group 2 (LRA), Mean ± SD (Range)	*P* Value^a^
Anesthesia time, min	3.6 ± 0.5 (2.6-4.3)	8.4 ± 2.0 (5.3-12.3)	**<.0001**
Intraoperative pain (NRS)	0.84 ± 1.46 (0-6)	0.64 ± 1.26 (0-5)	.65
Postoperative pain at 24 h (NRS)	3.0 ± 1.45 (1-6)	2.79 ± 1.71 (1-6)	.68
Recovery stay, h	9.45 ± 0.80 (7.86-10.99)	15.15 ± 8.41 (5.94-28.3)	**.004**

Abbreviations: LRA, locoregional anesthesia; NRS, Numeric Rating Scale; WALANT, wide-awake local anesthesia no tourniquet.

a Boldface indicates significance.

Postoperative recovery and discharge were based exclusively on standardized clinical criteria and were not influenced by organizational or logistical factors.

**Figure 1.**
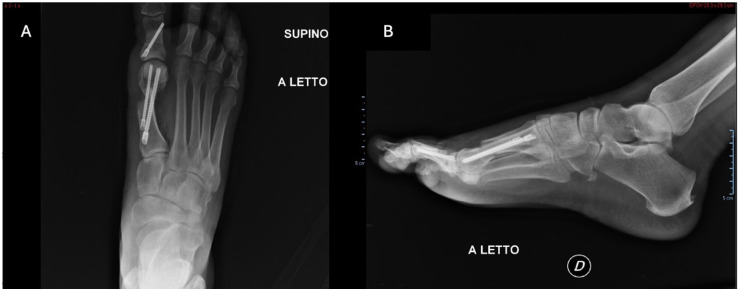
Postoperative radiographs obtained 1 month after MICA procedure: (A) anteroposterior (AP) view and (B) lateral view. MICA, minimally invasive chevron Akin.

**Figure 2.**
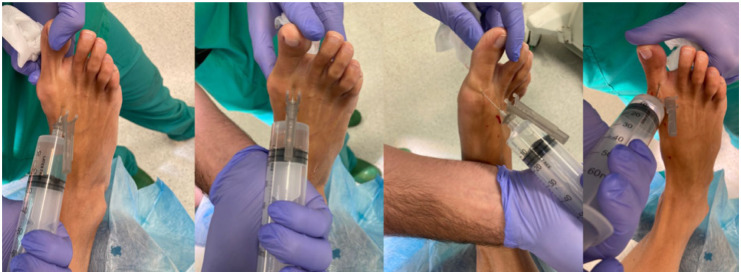
WALANT technique: The anesthetic solution is infiltrated directly along the planned incision and around the first metatarsal and proximal phalanx. WALANT, wide-awake local anesthesia no tourniquet.

No major intraoperative or postoperative complications were recorded in either group throughout the follow-up period (Figures 1 and 2).

## Discussion

This study shows that the WALANT technique provides comparable intraoperative and postoperative pain control to traditional LRA in MICA surgery for hallux valgus, while offering a significant reduction with the numbers available in anesthesia time and shorter hospital stay. All patients received a standardized postoperative analgesic regimen, and no differences in the type or amount of adjunctive analgesics with the numbers available, including opioids, were recorded between the WALANT and LRA groups.

No relevant differences in perioperative complications were observed between the WALANT and LRA groups with the numbers available. The results confirm the feasibility, safety, and efficiency of WALANT as an alternative anesthetic approach in minimally invasive foot surgery.^16,19,21-23^

Pain control was comparable between the 2 groups, with no statistically significant differences in intraoperative or 24-hour postoperative NRS scores with the numbers available. No cases of prolonged numbness or muscular weakness were observed, no postoperative nausea occurred, and no patients in either the WALANT or LRA group required hospital admission. All patients were discharged only after achieving adequate analgesic control, and no clinically relevant differences in postoperative recovery or pain control were observed between the WALANT and LRA groups. Postoperative discharge was determined according to standardized institutional criteria, including full recovery of baseline physiological status, adequate pain control, and safe ambulation, and was independent of the anesthetic technique used.

This demonstrates that WALANT can ensure adequate analgesia throughout the surgical procedure allowing patients to remain awake, comfortable, and fully cooperative during the procedure.^21,22,24^ The absence of conversion to another anesthetic technique and the lack of major complications further confirm its clinical reliability. WALANT was performed safely according to institutional protocol; cost analysis was not within the scope of this study.

These findings are consistent with previous published clinical trials on the use of WALANT in hand elective and trauma procedures, where similar levels of intraoperative values have been documented.^8,12^ The present study extends these results to elective foot and ankle surgery, where evidence is limited to a few published trials.^16,21^

This is the first randomized controlled clinical trial comparing LRA and WALANT in patients undergoing MICA, focusing on postoperative pain, anesthetic efficiency, and time to discharge. Its extension to foot and ankle surgery represents an evolution in the pursuit of minimally invasive and patient-centered surgical care.

A notable advantage observed in this study was the significantly shorter anesthesia time in the WALANT group with the numbers available (3.6 ± .5 minutes vs 8.4 ± 2.0 minutes). Anesthesia time was defined as the time required for anesthetic administration and onset of effect within the standardized perioperative workflow (in the operating room for WALANT and in the immediate preoperative area for LRA), and does not include overall preoperative waiting or hospital admission time.

This reduction reflects the procedural efficiency of direct local infiltration compared with the more complex execution of regional blocks, which require anatomical localization, ultrasound guidance, and postinjection latency. Although the absolute difference in anesthesia time between the 2 groups was small (4.8 minutes), both techniques demonstrated rapid onset, and even modest time reductions may contribute to improved operating room efficiency in high-volume settings.

Moreover, the hospital stay was shorter for patients undergoing WALANT procedures, most of whom were discharged the same day. The postoperative recovery time was related to institutional postoperative observation protocols rather than medical complications; all patients remained clinically stable during the entire recovery period. No patients in either group required additional analgesic medication during the postoperative recovery period. Discharge to home was allowed only after achievement of standard institutional criteria, including hemodynamic stability, adequate pain control with oral analgesia, absence of acute complications, and ability to mobilize safely with appropriate postoperative instructions. This time-saving factor may have meaningful implications for operating room turnover, scheduling flexibility, and outpatient workflow, particularly in high-volume centers or ambulatory settings.^16,25^

The use of 1% lidocaine with 8.4% sodium bicarbonate provides adequate sensory block. In our series, no intraoperative conversions or supplemental anesthesia was required, confirming the reliability of WALANT for this type of procedure. This supports the hypothesis that WALANT can provide analgesia equivalent to that of LRA while simplifying the anesthetic process.^16,21^ Moreover, the avoidance of regional anesthetic agents minimizes perioperative risks, mainly in older patients or those with comorbidities that may contraindicate regional anesthesia.^22,25^

Previous literature confirms these findings in lower limb procedures, including hallux valgus correction, metatarsal osteotomies, and Achilles tendon repair in which WALANT provides adequate anesthesia and hemostasis for distal extremity surgery with patient satisfaction and low complication rates.^21,25^ No major complications were recorded in either group, confirming the excellent safety profile of this approach.

This study has some limitations. The sample size is relatively small, reflecting its preliminary nature, and follow-up was limited to short-term outcomes. One important limitation of this study is that it represents a preliminary investigation and no a priori power calculation was performed.

Moreover, pain assessment was restricted to the first 24 postoperative hours, and long-term functional and satisfaction measures were not evaluated. Additionally, no radiologic outcomes were evaluated. The single-center design and involvement of a single surgical team may be a limit too.

Additional limitations should also be acknowledged. First, postoperative pain was not specifically assessed at the 4-hour time point, which may be clinically relevant considering the potentially different durations of action of the anesthetic techniques employed. Second, patient anxiety was not formally evaluated using a validated assessment tool, despite its possible influence on perceived pain and overall patient experience. Third, pain associated with needle insertion during anesthetic administration was not separately recorded, limiting the evaluation of patient discomfort related specifically to the injection procedure.

WALANT should be reserved for carefully selected patients. Relative contraindications include severe uncontrolled cardiovascular disease, significant coagulation disorders, severe peripheral vascular disease, active local infection, or inability to cooperate with the procedure. Patient selection must be individualized and the procedure performed in a setting equipped for appropriate perioperative monitoring and management of potential complications.

Future multicenter studies with larger cohorts and extended follow-up are warranted to confirm these findings and quantify the economic and organizational advantages of WALANT in foot surgery.

## Conclusions

In this preliminary randomized study, no statistically significant difference in intraoperative or postoperative pain was detected between WALANT and LRA with the numbers available. WALANT was associated with shorter anesthesia time and reduced recovery stay. The procedure was generally well tolerated, and no cases of anesthetic conversion were observed.

These findings suggest that WALANT may represent a feasible anesthetic approach for minimally invasive hallux valgus correction. However, larger studies are required to better define its safety profile and generalizability across different operators and institutions.

## Supplemental Material

sj-pdf-1-fao-10.1177_24730114261455303 – Supplemental material for Randomized Prospective Study Comparing Wide-Awake Local Anesthesia No Tourniquet (WALANT) and Locoregional Anesthesia in Minimally Invasive Hallux Valgus Surgery: Preliminary Results Supplemental material, sj-pdf-1-fao-10.1177_24730114261455303 for Randomized Prospective Study Comparing Wide-Awake Local Anesthesia No Tourniquet (WALANT) and Locoregional Anesthesia in Minimally Invasive Hallux Valgus Surgery: Preliminary Results by Daniele Marcolli, Alice Montagna, Ivan Pichierri, Tommaso Forin Valvecchi, Mirko Colombo, Riccardo Chierichini, Federico Alberto Grassi, Alessio Bernasconi and Pietro S. Randelli in Foot & Ankle Orthopaedics
